# Right ventricular function in dilated cardiomyopathy and ischemic heart disease: assessment with non-invasive imaging

**DOI:** 10.1007/s12471-015-0673-x

**Published:** 2015-03-06

**Authors:** S. Schalla, C. Jaarsma, S.C. Bekkers, J. Waltenberger, R. Dennert, H.J. Crijns, J. Wildberger, S. Heymans, H-P. Brunner-La Rocca

**Affiliations:** 1 Department of Cardiology, Maastricht University Medical Center, P. Debyelaan 25, P.O. Box 5800, 6202 AZ Maastricht, The Netherlands; 2 Department of Cardiology, University Hospital Muenster, Muenster, Germany; 3 Department of Radiology, Maastricht University Medical Center, Maastricht, The Netherlands

## Abstract

**Background:**

Dilated cardiomyopathy and ischaemic heart disease can both lead to right ventricular (RV) dysfunction. Direct comparisons of the two entities regarding RV size and function using state-of-the-art imaging techniques have not yet been performed. We aimed to determine RV function and volume in dilated cardiomyopathy and ischaemic heart disease in relation to left ventricular (LV) systolic and diastolic function and systolic pulmonary artery pressure.

**Methods and results:**

A well-characterised group (cardiac magnetic resonance imaging, echocardiography, coronary angiography and endomyocardial biopsy) of 46 patients with dilated cardiomyopathy was compared with LV ejection fraction (EF)-matched patients (*n* = 23) with ischaemic heart disease. Volumes and EF were determined with magnetic resonance imaging, diastolic LV function and pulmonary artery pressure with echocardiography.

After multivariable linear regression, four factors independently influenced RVEF (R^2^ = 0.51, *p* < 0.001): LVEF (*r* = 0.54, *p* < 0.001), ratio of peak early and peak atrial transmitral Doppler flow velocity as measure of LV filling pressure (*r* = − 0.52, *p* < 0.001) and tricuspid regurgitation flow velocity as measure of pulmonary artery pressure (r = − 0.38, *p* = 0.001). RVEF was significantly worse in patients with dilated cardiomyopathy compared with ischaemic heart disease: median 48 % (interquartile range (IQR) 37–55 %) versus 56 % (IQR 48–63 %), *p* < 0.05.

**Conclusions:**

In patients with dilated cardiomyopathy and ischaemic heart disease, RV function is determined by LV systolic and diastolic function, the underlying cause of LV dysfunction, and pulmonary artery pressure. It was demonstrated that RV function is more impaired in dilated cardiomyopathy.

## Introduction

Right ventricular (RV) dysfunction and dilatation are correlated to limited exercise capacity and poor outcome [1–4], but often neglected in the clinical setting [5, 6]. Dilated cardiomyopathy (DCM) and ischaemic heart disease (IHD) can both lead to RV dysfunction. Direct comparisons of the two entities with respect to RV size and function using state-of-the-art imaging techniques have not yet been performed. Such a comparison may help to better understand the underlying pathophysiology of RV dysfunction. Therefore, we determined RV function and volume in relation to left ventricular (LV) systolic and diastolic function, and pulmonary artery pressure in patients with DCM to assess the main mechanisms of RV dysfunction. In addition, after matching for LV ejection fraction (LVEF), patients with IHD due to infarction of the left coronary artery were also examined.

## Methods

### Study population

Two groups of patients were included in the study: patients with DCM from Maastricht University Medical Center and patients with IHD from our infarct database matched for LVEF.

Study subjects in the DCM group comprised 46 consecutive patients with DCM referred to our heart failure program. All underwent a standard diagnostic evaluation including electrocardiogram (ECG), coronary angiography, endomyocardial biopsy, echocardiography and cardiac magnetic resonance imaging (CMR). LV and RV volumes and systolic function were determined by CMR. LV diastolic function and RV systolic pressure were assessed with echocardiography as described later in the text. Patients with a history of myocardial infarction, history of cardiotoxic agents, significant coronary artery stenosis on coronary angiography, valvular heart disease on echocardiography as well as other known causes of impaired systolic function such as inflammation or infiltrative disorders on endomyocardial biopsy were excluded from the study. Patients with permanent pacemakers, rhythm other than sinus and significant chronic renal failure ( ≥ stage 3 kidney disease) were also excluded.

Study subjects in the IHD group were included from our infarct database matched for LVEF as measured by CMR. Thus, 23 patients with impaired systolic LV function due to chronic non-inferior myocardial infarction (infarction in left anterior descending (LAD) or left circumflex (LCX) coronary artery territory to avoid inclusion of patients with RV infarction) and without significant valvular disease were included. The study was approved by the local ethics committee.

### Magnetic resonance imaging

Patients were examined with a 1.5-T scanner (Gyroscan Intera,Philips Medical Systems, Best, the Netherlands). ECG-gated cine images were acquired for functional analysis using a steady-state free precession sequence (slice thickness 6 mm, gap 4 mm, repetition time/echo time 3.8/1.9 ms, flip angle 50°, field of view 350 mm, matrix 256 × 256, 22–25 phases per cardiac cycle). A breath-hold multislice T1-weighted three-dimensional inversion-recovery gradient-echo sequence (acquired/reconstructed slice thickness 12/6 mm, average repetition time/echo time 3.9/2.4 ms, multi-shot [50 profiles/shot] segmented partial echo readout, flip angle 15°, field of view 400 mm, matrix 256 × 256) to evaluate the presence of myocardial infarction of the left and right ventricle was used to acquire images 10 min after intravenous administration of 0.2 mmol/kg body weight gadolinium diethylenetriaminepentaacetic acid (Magnevist, Bayer, Germany). Inversion times were adjusted to null normal myocardium (200–280 ms).

CMR images were analysed by an investigator blinded to clinical and echocardiographic data. Endocardial and epicardial LV and endocardial RV contours were manually traced in end-diastolic and end-systolic phases on short-axis slices to determine LV and RV end-diastolic volume (EDV), end-systolic volume (ESV), ejection fraction (EF) and LV end-diastolic mass. Areas of late enhancement were visually assessed to confirm the presence and extent of infarction in the LAD or LCX territory as the reason for impaired LVEF in the IHD group.

### Echocardiography

Echocardiography was used to determine LV diastolic function, LA volume and peak tricuspid regurgitation (TI) Doppler velocity. Transthoracic echocardiograms were performed using dedicated systems (Sonos 5500 with S3 or IE33 with S5-1 transducers, Philips Medical Systems, Andover, MA, USA). Echocardiographic investigations were performed in the standard views, according to the recommendations of the American Society of Echocardiography [7]. LV diastolic function was assessed by measuring mitral (ratio of peak early and peak atrial transmitral Doppler flow velocity,E/A) and pulmonary vein flow velocities (ratio of peak systolic and diastolic pulmonary vein Doppler flow velocity,S/D), tissue Doppler flow velocities from the basal septal and lateral wall to calculate the ratio of early transmitral inflow and myocardial tissue velocity (E/e’) and left atrial (LA) volumes. Since we did not include patients with normal LV function, we used the E/A ratio as an easily obtainable measure of LV filling pressure. Peak tricuspid regurgitation (TI) Doppler flow velocity was used as a measure of pulmonary artery systolic pressure (PAP). Images were digitally stored and analysed off-line by an investigator blinded to CMR results and clinical data.

### Statistical analysis

Variables are expressed as percentage, mean ± standard deviation or median (interquartile range (IQR)) as appropriate. The Kolmogorov–Smirnov test was applied to test whether continuous variables were normally distributed. Group comparisons were performed by using the Pearson χ^2^ test, Student’s *t*-test or Mann–Whitney *U* test, as appropriate. For correlations, Pearson’s *r* was used. Finally, independent variables influencing RVEF were sought by using the multivariable linear regression model with a backward procedure (inclusion *p* < 0.05, exclusion *p* < 0.1).

## Results

### Clinical characteristics of patients with DCM and IHD

The clinical characteristics of the DCM and IHD patients are summarised in Table 1. Patients in the IHD group were older and less often treated with diuretics. All other parameters were not significantly different between the groups.

**Table 1:** Clinical, cardiac magnetic resonance imaging and echocardiographic characteristics of the patients with dilated cardiomyopathy (DCM) and ischaemic heart disease (IHD)

Characteristic	DCM (*n* = 46)	IHD (*n* = 23)	*p* Value
Age, years	49 ± 14	59 ± 16	< 0.01
Male, *n* (%)	27 (59 %)	18 (78 %)	0.18
Body surface area, m^2^	1.92 ± 0.25	1.97 ± 0.19	0.28
Dyspnoea			0.48
NYHA 1/2, *n* (%)	38 (83 %)	21 (91 %)	
NYHA 3/4, *n* (%)	8 (17 %)	2 (9 %)	
Duration HF, months	4 (1–18)	3 (0–19)	0.98
Diabetes mellitus			0.62
Type 1, *n* (%)	1 (2 %)	0 (0 %)	
Type 2, *n* (%)	4 (9 %)	1 (4 %)	
COPD, *n* (%)	6 (13 %)	2 (9 %)	0.71
Hypertension, *n* (%)	9 (18 %)	8 (35 %)	0.24
Systolic BP	125 ± 20	123 ± 19	0.80
Diastolic BP	75 ± 12	74 ± 10	0.75
Heart rate, beats/min	77 ± 14	73 ± 13	0.32
PR duration, ms	150 ± 57	157 ± 60	0.66
QRS duration, ms	111 ± 31	92 ± 43	0.10
LBBB, *n* (%)	16 (35 %)	4 (17 %)	0.17
RBBB, *n* (%)	0 (0 %)	2 (9 %)	0.11
Creatinine, µmol/l	92 ± 27	95 ± 45	0.74
Beta-blocker	36 (78 %)	18 (78 %)	0.34
ACE-inhibitor or AT-II-receptor blocker	40 (87 %)	21 (91 %)	0.20
Diuretic	32 (70 %)	9 (39 %)	0.03
Aldosterone antagonist	12 (26 %)	4 (17 %)	0.28
Calcium channel blocker	0 (0 %)	1 (4 %)	0.13
RV EDV, ml/m^2^	78 (65–92)	71 (63–78)	0.03
RV ESV, ml/m^2^	41 (35–51)	32 (24–39)	0.03
RV SV, ml/m^2^	35 (28–43)	38 (29–43)	0.98
RVEF, %	48 (37–55)	56 (48–63)	0.05
LV EDV, ml/m^2^	120 (96–158)	131 (101–165)	0.79
LV ESV, ml/m^2^	82 (64–117)	85 (66–128)	0.63
LV SV, ml/m^2^	39 (29–46)	38 (34–43)	0.75
LVEF, %	31 (22–40)	34 (18–39)	0.77
LV mass, g/m^2^	75 (62–84)	68 (62–86)	0.86
RA volume, ml/m^2^	20 (16–32)	22 (17–26)	0.43
LA volume, ml/m^2^	32 (26–53)	37 (28–46)	0.86
E max velocity, cm/s	71 (55–82)	68 (53–98)	0.67
E/A	1.00 (0.70–1.40)	0.96 (0.63–1.78)	0.99
dt E-top ms	170 (130–205)	160 (130–220)	0.68
S/D	1.04 (0.81–1.33)	1.00 (0.62–1.45)	0.79
E/e’ IVS	10.0 (8.0–13.0)	6.5 (4.9–7.5)	< 0.001
E/e’	7.7 (5.4–9.7)	8.4 (6.6–11.5)	0.31
TI peak velocity, m/s	2.13 (1.86–2.44)	2.42 (2.02–3.03)	0.04

Values represent mean ± standard deviation, median (interquartile range) or n, numbers of patients (%)

*DCM* dilated cardiomyopathy, *IHD* ischaemic heart disease, *NYHA* New York Heart Association class, *COPD* chronic obstructive pulmonary disease, *HF* heart failure, *BP* blood pressure, *LBBB* left bundle branch block, *RBBB* right bundle branch block, *ACE* angiotensin converting enzyme, *AT II* angiotensin II, *RV* right ventricular, *LV* left ventricular, *EDV* end-diastolic volume, *ESV* end-systolic volume, *SV* stroke volume, *EF* ejection fraction, *PASP* pulmonary artery systolic pressure, *RA* right atrial, *LA* left atrial, *E* peak early transmitral Doppler flow velocity, *E/A* ratio of peak early and peak atrial transmitral Doppler flow velocity, *dt E-top* deceleration time of peak early transmitral Doppler flow signal, *S/D* ratio of peak systolic and diastolic pulmonary vein Doppler flow velocity, *E/e’ IVS* ratio of peak early transmitral Doppler flow velocity and peak early diastolic tissue Doppler flow velocity from the basal septal left ventricular wall, *E/e’* ratio of peak early transmitral Doppler flow velocity and peak early diastolic tissue Doppler flow velocity from the basal lateral left ventricular wall, *TI* peak tricuspid regurgitation Doppler flow velocity

CMR image analysis was possible in all patients. In three patients, however, image quality on echocardiography was not sufficient to obtain reliable pulmonary vein flow Doppler signals. In two of them, image quality was also not sufficient to obtain tissue Doppler signals, but the E/A ratio could be determined in all patients.

**Fig. 1:**
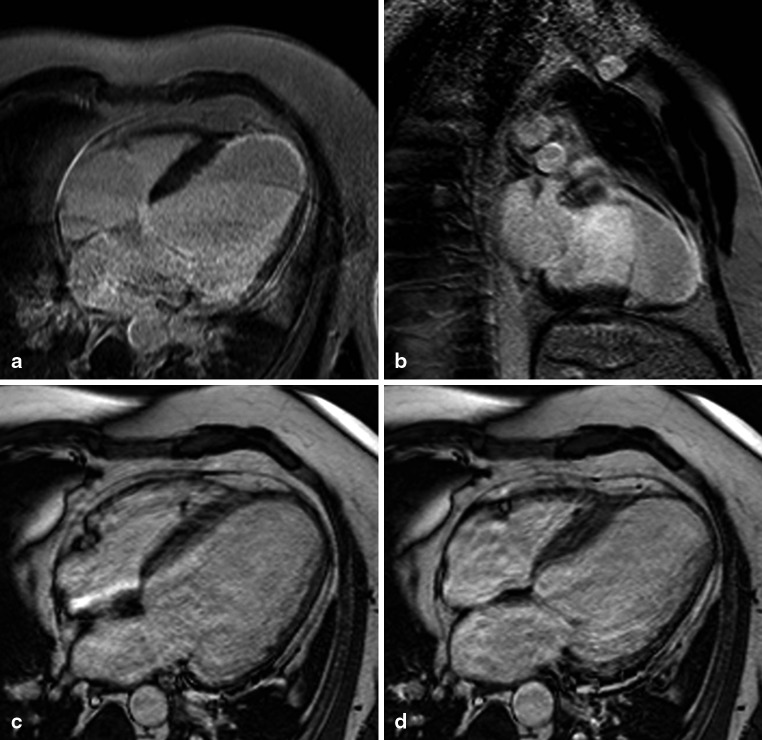
Late gadolinium enhancement (**a**, **b**) and cine (four-chamber view in end-diastole (**c**) and end-systole (**d**)) magnetic resonance images of a patient with a large myocardial infarction (left ventricular ejection fraction 15 %) of the left anterior descending coronary artery (right ventricular ejection fraction 56 %)

**Fig. 2:**
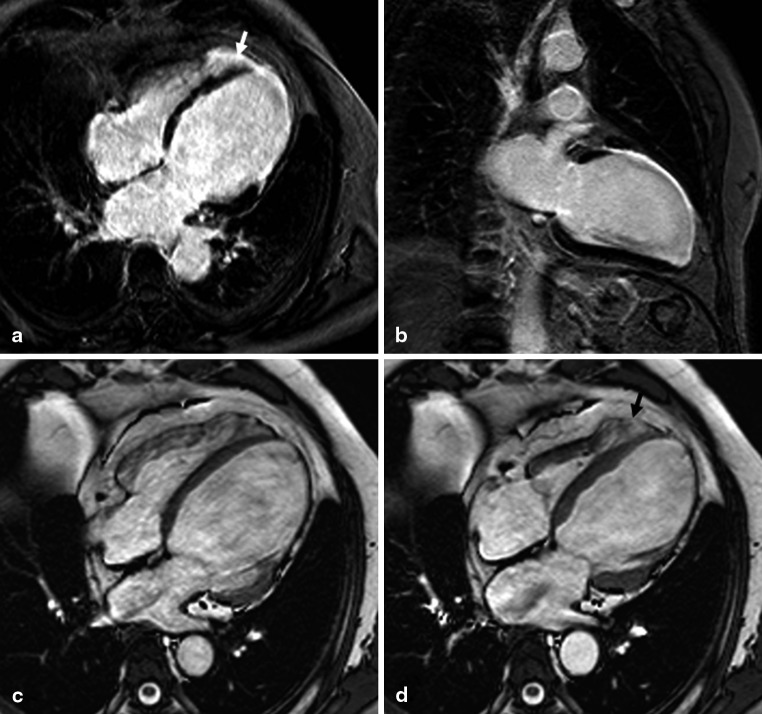
Late gadolinium enhancement (four-chamber view (**a**), 2 chamber view (**b**)) and cine (four-chamber view in end-diastole (**c**) and end-systole (**d**)) magnetic resonance images of a patient with a large myocardial infarction (left ventricular ejection fraction 22 %) of the left anterior descending coronary artery, continuing from the left ventricular apex to the right ventricle (right ventricular ejection fraction 57 %) (*white arrow*) A corresponding akinetic region of the right ventricular apex can be depicted on the end-systolic cine image (D, *black arrow*)

A total of 21 patients with IHD had infarcts in the LAD territory and 2 patients in the LAD and LCX territory (Fig. 1). Infarction of the RV free wall was recognised in only one patient of the IHD group: a small area of late enhancement of the RV apex was visible on CMR images. This patient had a transmural infarction in the LAD territory showing late enhancement from the anterior wall to the apex of the LV continuing to the RV apex (Fig. 2). None of the patients with DCM had focal fibrosis of the RV free wall myocardium on CMR late enhancement images.

### RV volume and function

#### Differences between patients with DCM and IHD

**Fig. 3:**
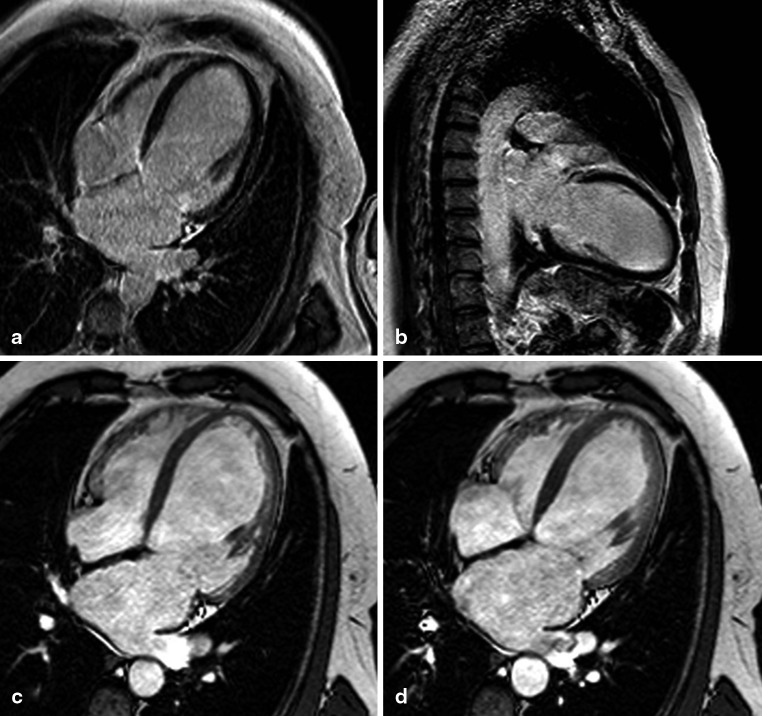
Late gadolinium enhancement (**a**, **b**) and cine (four-chamber view in end-diastole (**c**) and end-systole (**d**)) magnetic resonance images of a patient with idiopathic dilated cardiomyopathy (left ventricular ejection fraction 24 %, right ventricular ejection fraction 45 %) The left ventricular systolic function of this patient is slightly better than that of the patients with ischaemic heart disease from Figs. 1 and 2 while the right ventricular function is more impaired. Areas of late enhancement are not present

**Fig. 4:**
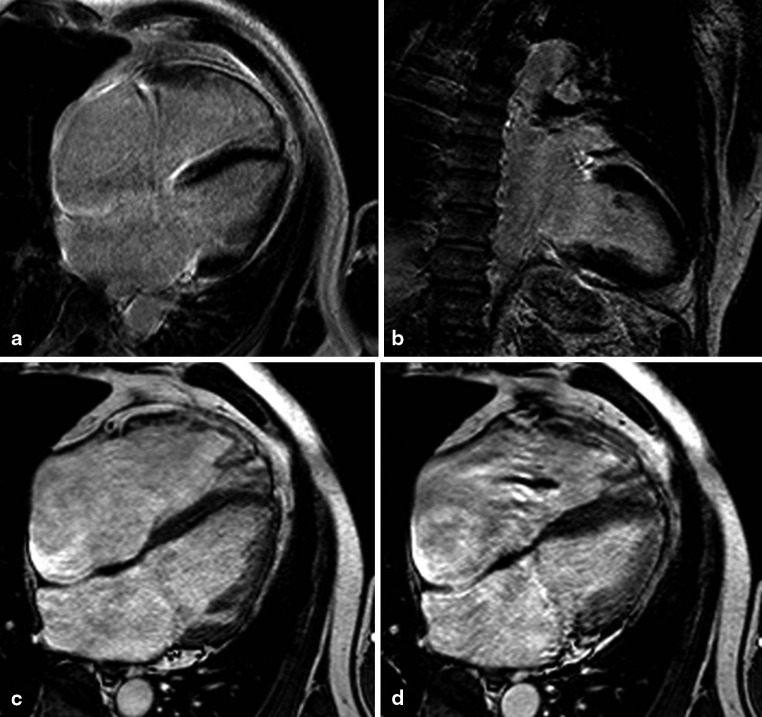
Late gadolinium enhancement (**a**, **b**) and cine (four-chamber view in end-diastole (**c**) and end-systole (**d**)) magnetic resonance images of a patient with idiopathic dilated cardiomyopathy with more pronounced dilation of the right ventricle (left ventricular ejection fraction 36 %, end-diastolic volume 211 ml; right ventricular ejection fraction 23 %, end-diastolic volume 269 ml) Although elevated left ventricular filling pressure and TI velocity were present, the degree of right ventricular dilation is out of proportion, possibly suggesting an active unknown process affecting the right ventricle more than the left ventricle

The RVEF was more impaired and RV size larger in patients with DCM in comparison with patients with IHD (Figs. 3 and 4) despite similar LV systolic and diastolic dysfunction. There was an isolated increase in E/e’ of the septum in DCM patients, but an opposite trend laterally and no further differences were seen in diastolic parameters between the groups, suggesting no difference in diastolic LV function. LV EDV and ESV were not different between the groups. All CMR and echocardiography parameters are summarised in Table 1.

#### Parameters influencing RV function

As depicted in Table 2, the RVEF was significantly correlated with LVEF and the volumes of all four cardiac chambers. Furthermore, parameters of LV diastolic function negatively correlated with RV function. Also, the TI velocity as measure of PAP was negatively correlated with RVEF. Finally, age and heart rate showed significant correlations with RVEF. RVEF was higher in women compared with men (55 ± 10 versus 44 ± 13 %, *p* = 0.001). Other clinical parameters had no influence on RVEF (data not shown). Overall, there were no significant differences in these correlations between patients with DCM and IHD (data not shown).

**Table 2:** Correlations of clinical, cardiac magnetic resonance imaging (CMR) and echocardiographic characteristics with right ventricular ejection fraction as measured with CMR

Variable	Correlation	*p* Value
Age	0.29	0.02
Systolic BP	0.13	0.28
HR	− 0.46	< 0.001
LVEF	0.54	< 0.001
LV EDV	− 0.29	0.02
RV EDV	− 0.50	< 0.001
LA volume	− 0.41	< 0.001
RA volume	− 0.37	< 0.001
E/A ratio	− 0.52	< 0.001
S/D ratio	0.48	< 0.001
TI velocity	− 0.38	0.001

*BP* blood pressure, *HR* heart rate, *LV* left ventricular, *RV* right ventricular, *EF* ejection fraction, *EDV* end-diastolic volume, *LA* left atrial, *RA* right atrial, *E/A* ratio of peak early and peak atrial transmitral Doppler flow velocity, *S/D* ratio of peak systolic and diastolic pulmonary vein Doppler flow velocity, *TI* peak tricuspid regurgitation Doppler flow velocity

Multivariable analysis revealed that the RV function (i.e. RVEF) was correlated with LVEF, DCM compared with IHD, E/A ratio as measure of LV filling pressure, and TI velocity as measure of PAP as depicted in Table 3. Although the relationship with TI velocity was of borderline significance, it was in addition to a measure of LV filling pressure, e.g. a possible active process in addition to passive elevation of PAP due to elevated LV filling pressures.

**Table 3:** Regression analysis testing the association between right ventricular ejection fraction and various potential predictors in the entire study population (dilated cardiomyopathy and ischaemic heart disease)

Variable	Regression coefficient	*p* Value
LVEF (per %)	0.50	< 0.0001
DCM versus IHD	9.41	0.0005
E/A ratio (per unit)	− 3.60	0.02
TI velocity (per m/s)	− 4.84	0.06

(R^2 ^= 0.51)

*DCM* dilated cardiomyopathy, *IHD* ischaemic heart disease, *LVEF* left ventricular ejection fraction, *E/A* ratio of peak early and peak atrial transmitral Doppler flow velocity, *TI* peak tricuspid regurgitation Doppler flow velocity

## Discussion

In the current study, the non-invasive imaging techniques CMR and echocardiography were applied in patients with DCM and IHD without RV free wall infarction, to evaluate RV function and volumes. RV systolic function was influenced by different factors including the underlying disease process, i.e. the presence of DCM, systolic and diastolic function of the LV and elevation of the PAP.

In ST-segment elevation myocardial infarction (STEMI) patients with cardiogenic shock, RV dysfunction had been identified as a prognostic parameter [8]. Between patients with and without RV dysfunction, no significant differences in infarct-related artery and infarct location were observed. RV dysfunction in patients in whom the right coronary artery (RCA) was not the infarct-related artery was also present. This suggests that not only direct RV infarction causes RV dysfunction but also mechanisms such as reduced blood supply of the septum, low RV preload due to low output and diminished contribution of LV contraction to RV systole [8]. RV function may depend on LV septal contractile contribution transmitted through systolic ventricular interaction [9–11] and the septum itself may contribute to the systolic function of both ventricles [12]. RV function was experimentally as much impaired with septal ligation as with RCA ligation [13]. Our results, however, suggest that other factors such as elevated LV filling pressure and PAP are of additional importance to explain reduced RV function.

RV infarction detected by late enhancement CMR and RV systolic function were outcome predictors in a general population of STEMI patients [14]. In inferior STEMI, RV systolic function was related to the presence and extent of RV infarction, while in anterior STEMI it was related to LV systolic dysfunction. When imaging more early after infarction, RV infarction was not related to prognosis [15]. This discrepancy could be explained by the fact that RV function may recover to a greater extent than LV function, depending on recovery of septal function [9]. Thus, global RV performance recovered within days after infarction, regardless of artery patency [16]. Moreover, global RV performance improved greatly even in chronic RCA occlusion, despite persistent severe RV free wall dysfunction [17]. To exclude the influence of direct RV free wall infarct on RV function, we included only patients with infarcts of the left coronary artery territory (LAD and LCX infarct). To our knowledge, this has not been applied in previous comparative studies of patients with DCM and IHD. We observed a small extension of infarct from the left to the RV myocardium in one patient only, which is in some contrast to a recent CMR study in 20 patients with reperfused proximal LAD occlusion where a small RV infarct was observed in 40 % of the patients [18]. They found a relatively large area of the RV at risk for necrosis, although the resulting final RV infarction size was small. A possible explanation for the discrepancy between this and our findings is that only a small portion of our patients had transmural infarcts of the whole septum.

The underlying process also determines RV function. Patients with DCM experienced larger RV volumes and more severe impairment of RV function. In contrast to previous suggestions [19, 20], assessing systolic RV function alone is not sufficient to distinguish between DCM and IHD in individual patients. Few studies have directly compared the RV function in patients with DCM and IHD. Our findings are supported by some earlier studies where patients with DCM showed more severe RV dysfunction than patients with IHD using radionuclide angiography, thermodilution and invasive RV angiography [19–21]. However, these findings have not been uniform. By applying tissue Doppler imaging, the RV dysfunction was more pronounced in patients with IHD than DCM in an echocardiographic study [22]. Since only two patients had inferior wall infarction based on ECG criteria, the authors concluded that this worse RV function had not been due to more infarcted RV myocardium. However, patients were not sufficiently matched with respect to other factors potentially influencing RV function. Thus, patients with IHD exhibited more severe LV diastolic dysfunction and higher PAP. Such a difference in PAP and diastolic function was not present in our patient groups, but PAP and diastolic function were independently related to RV dysfunction. Moreover, assessment of RV dimensions and systolic function by echocardiography has important limitations. In the present study, we therefore used the current reference standard CMR to assess RV volumes and EF [6, 23, 24].

Elevated LV filling pressure, which leads to passive elevation of PAP, was one of the factors related to RV dysfunction in the current study. Since the prognosis of patients with impaired RV function is worse in comparison with patients with impaired systolic LV function only [25, 26], assessment of RV function and possibly more aggressive treatment of elevated LV filling pressure as suggested by Stevenson et al. [27] might be important, irrespective of the underlying cause. An additional active component of elevated pulmonary artery pressure, i.e. out-of-proportion pulmonary hypertension leading to fixed pulmonary hypertension, may also have contributed to worsening of RV function since elevated PAP was related to RV dysfunction independently of LV filling pressure.

The current study has some limitations. Patients in the IHD group were older and less often treated with diuretics. Older age had a negative influence on RV function. However, although the patients in the IHD group were older, they showed a less impaired RV function than the patients from the DCM group. Thus, not matching for age did not result in a relevant bias. Treatment with diuretics can lower LV filling pressure and PAP and might have had an influence on the lower TI peak velocity measured in patients with DCM [28]. In contrast to IHD, DCM usually comprises a heterogeneous group of diseases in many studies. However, with a comprehensive diagnostic routine including endomyocardial biopsy, imaging and blood tests, certain diseases such as acute myocarditis, infiltrative and storage diseases were excluded from our study to make the DCM study population as uniform as possible. Regional RV wall motion as measured by, e.g. speckle tracking was not assessed. Early stages of dyssynchronicity between the right and left ventricle were therefore not detected. Other potential mechanisms were also not addressed, e.g. interventricular interaction and effects of changes of geometry. Finally, this was a diagnostic study. However, the study revealed different potential mechanisms of RV dysfunction that may be therapeutically influenced. It is important to evaluate RV function, especially in DCM, and it may be necessary to treat elevated filling pressure and PAP more aggressively or with new therapeutic strategies such as phosphodiesterase inhibitors to protect the right ventricle.

In conclusion, RV systolic function was influenced by different factors including the underlying disease process, i.e. the presence of DCM, systolic and diastolic function of the LV and elevation of PAP. A better understanding of these mechanisms may help to define therapeutic targets for future studies in these patients with RV dysfunction known to have a poor outcome.
